# The Association of Percent Body Fat and Lean Mass With HbA_1c_ in US Adults

**DOI:** 10.1210/js.2017-00046

**Published:** 2017-04-18

**Authors:** Julie K. Bower, Rachel J. Meadows, Meredith C. Foster, Randi E. Foraker, Abigail B. Shoben

**Affiliations:** 1 The Ohio State University College of Public Health, Columbus, Ohio; 2 The Ohio State University College of Medicine, Columbus, Ohio 43210; 3 Signature Programme in Health Services and Systems Research, Duke-National University of Singapore Medical School, Singapore 169857

## Abstract

**Context:**

Body fat and body composition distribution patterns affect diabetes risk and glycemic control, but most studies use proxy measures (*e.g.,* body mass index).

**Objective:**

This study examined the association of percent body fat and lean mass with glycated hemoglobin (HbA_1c_) in US adults.

**Design:**

The National Health and Nutrition Examination Survey (NHANES) is a program of cross-sectional studies that enroll nationally representative samples of the US civilian noninstitutionalized population.

**Setting:**

NHANES is designed to assess the health status of adults and children throughout the United States.

**Participants:**

This study included 11,125 participants aged 18 to 69 years from the 1999 through 2006 NHANES, comprising 846 persons with diagnosed diabetes and 10,125 without diabetes.

**Main Outcome Measures:**

Total and abdominal (trunk) percent body fat and lean mass were measured using dual-energy x-ray absorptiometry. Linear and logistic regression analyses were used to examine their association with HbA_1c_.

**Results:**

Among those without diagnosed diabetes, total and trunk percent body fat, as well as trunk and total lean mass, were strongly associated with elevated HbA_1c_; odds ratios per 5% increment for the association of percent body fat with HbA_1c_ >5.7% (39 mmol/mol) ranged from 1.60 to 2.01 across age and sex categories. Among adults with diabetes, higher total percent fat was associated with higher HbA_1c_ in males age <40 years and higher trunk fat was associated with higher HbA_1c_ in females across age categories.

**Conclusions:**

Lifestyle interventions to lower HbA_1c_ should consider targeting both weight loss and body composition.

## Teaser

This study examines the independent association of percent body fat and lean mass with HbA_1c_ in a nationally representative sample of US adults.

Obesity is a well-established risk factor for diabetes and related metabolic disorders [1–3]. Abdominal (trunk) and total adiposity are strongly associated with insulin resistance, development of diabetes, and glycemic control in patients with established disease [4, 5]. Weight loss remains a key component of diabetes prevention and management because of the known effect of adiposity on insulin sensitivity and resistance [6]. Although both muscle (lean mass) and adipose tissue serve important metabolic functions, most studies of obesity and diabetes use proxy measures for overall or abdominal obesity such as body mass index (BMI) or waist-hip ratio without accounting for the composition of that mass [5, 7–10].

Current guidelines for disease management in diabetes patients and for prevention of diabetes among high-risk populations includes 150 minutes of moderate-intensity activity and weight loss of at least 7% of body weight for persons who are overweight or obese [11]. Self-management guidelines for persons with diabetes also endorse resistance training at least twice per week for patients without contraindications in addition to the minimum of 150 minutes of moderate-intensity aerobic activity [11], though this recommendation is less widely known and disseminated. Although decreasing overall body weight improves hyperglycemia, controlled laboratory studies clearly demonstrate the importance of the composition of that mass. That is, a lower proportion of overall mass that is fat or adipose tissue and a higher proportion that is lean mass are independently associated with improved glucose metabolism and *β* cell function [12–16].

Prior evidence demonstrates that a combination of aerobic and resistance training improves glycated hemoglobin (HbA_1c_) greater than either alone, although resistance training is commonly neglected from lifestyle interventions aimed at addressing hyperglycemia [12, 14, 17]. The role of lean mass and body fat distribution in glucose metabolism and insulin resistance is well studied in controlled laboratory settings [12, 14, 18], but fewer data are available from population-based studies. Dual-energy x-ray absorptiometry (DXA) is widely considered a preferred method for assessing body composition [19, 20]. Thus, the aim of this study was to quantify the association of body fat and lean mass (total and abdominal) with HbA_1c_—an indicator of glucose control in those with diabetes and a risk marker in nondiabetic populations—in a representative sample of the US adult population that underwent a DXA scan.

## 1. Materials and Methods

### A. Study Population

We analyzed data from the 1999 through 2006 National Health and Nutrition Examination Survey (NHANES). The NHANES are cross-sectional, nationally representative surveys of the US civilian noninstitutionalized population conducted by the National Center for Health Statistics [21]. This study included 11,125 nonpregnant participants 18 to 69 years of age from the 1999 through 2006 NHANES; participants with missing body composition or HbA_1c_ data were excluded. A human subjects review board approved data collection procedures and written informed consent was obtained from all study participants [22].

### B. Measurements

#### B-1. Body fat and lean mass

Percent body fat and lean mass (total and abdominal/trunk only) were determined using DXA. DXA scans were performed in the supine position using a Hologic QDR-4500A fan-beam densitometer (Hologic, Inc., Bedford, MA) that was calibrated daily using a spine phantom and one to three times weekly using a whole-body phantom, and analyzed using Hologic Discovery software (version 12.4) [23]. Percent body fat and lean mass relative to total body mass was calculated by dividing the total weight of lean mass (excluding bone mineral content) in the whole body (for total percent body fat and lean mass) and in the abdominal region (for trunk percent fat and lean mass) by the participant’s total weight and multiplying by 100 to produce a percentage.

#### B-2. HbA_1c_ and other variables of interest

HbA_1c_ measurements were obtained using high-performance liquid chromatography, standardized to the Diabetes Control and Complication Trial assay [24]. Height and weight were directly measured using standard procedures performed by trained NHANES personnel. BMI was calculated as weight in kilograms divided by height in meters squared. Demographic information (including age, sex, and race/ethnicity) was self-reported by study participants. A history of diabetes was defined as self-reported diagnosis of diabetes or current use of insulin.

### C. Statistical Analysis

Analyses incorporated the NHANES sample weights using standard methods to account for the complex, multistage, probability sampling design. Sample weights incorporated adjustment for unequal probability of selection, nonresponse, and coverage errors; variance units were specified to account for stratified sampling and clustering [21]. The Taylor series linearization method was used for variance estimation. Demographic, HbA_1c_, and measures of body composition were summarized in adults and stratified by diabetes history, sex, and age. Correlations of percent body fat (total and trunk) and lean mass (total and trunk) with anthropometry-based body measurements (BMI, height) were evaluated using Spearman correlation coefficients. Associations of four body composition measures with HbA_1c_ as a continuous outcome were evaluated using multivariable linear regression (total percent body fat, trunk percent body fat, total percent lean mass, and trunk percent lean mass). Additionally, logistic regression was used to assess the association of body composition measures with elevated HbA_1c_ (≥5.7% or 39 mmol/mol) in the subgroup without a diagnosis of diabetes or use of insulin. Effect estimates are presented with 95% confidence intervals (CIs). *P* < 0.05 was considered statistically significant. Because of our *a priori*–specified hypotheses based on the current literature, we did not account for multiple comparisons.

Measures of association for percent body fat and lean mass are presented per 5% higher increment because a 5% increase in percent body fat is approximately equivalent to a one-category increase in commonly used body fat percentage categories [25]. Models were stratified by diagnosed diabetes status to examine associations with HbA_1c_ separately in populations where persons were likely receiving treatment of hyperglycemia that would alter HbA_1c_ vs those that were treatment-naïve. Additionally, models were further stratified by sex and age (<40 years, ≥40 years) to account for known differences in body composition in males vs females and younger vs older adults.

All models were adjusted for age as a continuous variable, race/ethnicity, and height. Multiple imputation was applied to account for missing DXA data to address potential bias resulting from nonrandom missing data by age, BMI, weight, height, and other key participant characteristics using sequential regression multivariate imputation [26]. Five complete data files that contained both the nonmissing and imputed values (generated using sequential multivariate imputation) were created. Analyses were performed using Stata Statistical Software, release 13.1 (StataCorp LP, College Station, TX).

## 2. Results

The study population included 846 adults with diagnosed diabetes and 10,125 adults with no clinical history of diabetes. Adults without diagnosed diabetes were 41 years of age on average, and the population was 50% male and 71% non-Hispanic white. The mean BMI in adults without diagnosed diabetes was 27.8 kg/m^2^ with a total percent body fat of 33%, trunk percent body fat of 33%, total percent lean mass of 64%, and trunk percent lean mass of 66%. Adults with diagnosed diabetes were 53 years of age on average, 51% were men, and 58% were non-Hispanic white. This group also had a mean BMI of 32.9 kg/m^2^, total percent body fat of 37%, trunk percent fat of 38%, total percent lean mass of 61%, and trunk percent lean mass of 60%. The mean values for all body composition measures differed significantly between those with diabetes and those without (*P* < 0.001 for all comparisons). Population characteristics further stratified by sex and age groups are summarized in Table 1.

**Table 1.** **Selected Participant Characteristics Stratified by Diagnosed Diabetes Status, Sex, and Age**

	**No History of Diabetes**				**Diagnosed Diabetes**			
	**Male**		**Female**		**Male**		**Female**	
	**20-39 y**	**40-69 y**	**20-39 y**	**40-69 y**	**20-39 y**	**40-69 y**	**20-39 y**	**40-69 y**
Unweighted, N	2779	2526	2560	2588	35	385	50	376
Age (y)	28.7 ± 0.2	51.5 ± 0.5	28.9 ± 0.2	51.8 ± 0.2	34.4 ± 0.8	55.1 ± 0.5	32.1 ± 0.8	56.6 ± 0.6
Race/ethnicity (%)								
Non-Hispanic white	64.7	78.7	64.3	75.7	59.1	64.1	38.9	53.3
Non-Hispanic black	11.1	8.8	13.0	9.8	14.5	13.5	25.7	18.9
Mexican American	11.6	5.2	9.2	4.7	4.4	8.2	13.1	9.8
Other or multiracial	12.6	7.4	13.5	9.9	21.9	14.2	22.3	18.0
Body mass index (kg/m^2^)	26.9 ± 0.1	28.5 ± 0.2	27.0 ± 0.2	28.7 ± 0.2	33.7 ± 1.5	31.9 ± 0.6	33.2 ± 1.7	33.8 ± 0.5
Waist circumference (cm)	93.9 ± 0.4	102.2 ± 0.4	88.3 ± 0.5	94.4 ± 0.5	114.7 ± 3.8	110.6 ± 1.4	103.2 ± 3.5	108.4 ± 0.9
Total fat (mean %)	25.5 ± 0.2	28.8 ± 0.2	37.5 ± 0.2	40.5 ± 0.2	31.5 ± 1.1	31.3 ± 0.5	40.2 ± 1.3	43.1 ± 0.4
Trunk fat (mean %)	25.7 ± 0.2	30.4 ± 0.2	35.1 ± 0.3	39.1 ± 0.2	33.6 ± 1.3	33.8 ± 0.6	40.5 ± 1.6	43.7 ± 0.4
Total lean mass (mean %)	71.9 ± 0.2	68.7 ± 0.2	59.9 ± 0.1	57.1 ± 0.2	66.4 ± 1.0	66.4 ± 0.5	57.6 ± 1.3	54.8 ± 0.4
Trunk lean mass (mean %)	72.5 ± 0.2	68.0 ± 0.2	63.1 ± 0.3	59.4 ± 0.2	65.0 ± 1.2	64.8 ± 0.5	58.0 ± 1.6	55.0 ± 0.4
HbA_1c_ (mean %)	5.1 ± 0.01	5.4 ± 0.02	5.1 ± 0.01	5.4 ± 0.02	8.3 ± 0.4	7.5 ± 0.1	8.6 ± 0.4	7.4 ± 0.1
Undiagnosed diabetes (%)^*a*^	0.4	3.1	0.4	1.7	n/a	n/a	n/a	n/a
Prediabetes (%)*^b^*	4.6	16.9	3.7	17.8	n/a	n/a	n/a	n/a

Data are weighted means ± standard error or proportions.

^a^ HbA_1c_ ≥ 6.5%.

^b^ HbA_1c_ 5.7% to 6.4%.

The Spearman correlations between BMI and percent total body fat and percent trunk fat were 0.61 and 0.73, respectively (both *P* < 0.0001). BMI was positively correlated with total lean mass and trunk lean mass (0.49 and 0.50, respectively). Height was negatively correlated with percent total body fat (−0.51, *P* < 0.0001) and percent trunk body fat (−0.41, *P* < 0.0001), and positively correlated with total lean mass (0.76, *P* < 0.0001) and trunk lean mass (0.73, *P* < 0.0001).

Among those without diagnosed diabetes, higher percent total body fat and trunk body fat were strongly positively associated with HbA_1c_ across all population subgroups (Table 2). Each 5% increase in percent total body fat was associated with 0.04% to 0.11% points higher HbA_1c_ across groups. For trunk percent fat in those without diabetes, each 5% increase was associated with 0.04% to 0.07% points of higher HbA_1c_. Lean mass was consistently inversely associated with HbA_1c_ across age and sex categories (Table 2). The association between total and percent fat, as well as total lean mass and HbA1c, differed by sex (*P* interaction < 0.05). The association of total percent fat as well as total and trunk lean mass with HbA_1c_ differed by age group (*P* interaction < 0.01).

**Table 2.** **Adjusted *β*-Coefficients (95% CI) for the Association of Percent Body Fat and Lean Mass (Total and Trunk per 5%) with HbA_1c_, NHANES 1999-2006**

	**Body Fat**				**Lean Mass**			
	**Total**	***P* Value**	**Trunk**	***P* Value**	**Total**	***P* Value**	**Trunk**	***P* Value**
No diabetes								
Male, y								
<40	0.04 (0.02–0.06)	<0.001	0.04 (0.02–0.05)	<0.001	−0.04 (−0.06 to −0.03)	<0.001	−0.04 (−0.05 to −0.02)	<0.001
≥40	0.11 (0.08–0.14)	<0.001	0.09 (0.07–0.12)	<0.001	−0.12 (−0.15 to −0.08)	<0.001	−0.10 (−0.12 to −0.07)	<0.001
Female, y								
<40	0.04 (0.03–0.05)	<0.001	0.04 (0.03–0.05)	<0.001	−0.04 (−0.05 to −0.04)	<0.001	−0.04 (−0.05 to −0.03)	<0.001
≥40	0.07 (0.06–0.08)	<0.001	0.07 (0.06–0.08)	<0.001	−0.07 (−0.08 to −0.06)	<0.001	−0.07 (−0.08 to −0.06)	<0.001
Diagnosed diabetes								
Male, y								
<40	0.04 (0.01–0.07)	0.01	0.03 (0.00–0.06)	0.06	−0.04 (−0.07 to −0.01)	0.01	−0.03 (−0.06 to 0.002)	0.1
≥40	0.03 (−0.09 to 0.16)	0.60	0.09 (−0.01 to 0.19)	0.09	−0.02 (−0.15 to 0.12)	0.8	−0.08 (−0.19 to 0.17)	0.1
Female, y								
<40	0.02 (−0.02 to 0.05)	0.39	0.05 (0.01–0.08)	0.006	−0.005 (−0.04 to 0.27)	0.8	−0.09 (−0.07 to 0.10)	0.7
≥40	0.03 (−0.06 to 0.13)	0.49	0.11 (0.06–0.17)	<0.001	−0.04 (−0.14 to 0.06)	0.4	−0.05 (−0.08 to −0.01)	0.008

Adjusted for age, race/ethnicity, and height.

Associations were less consistent in the subpopulation with diagnosed diabetes. In men, total percent body fat was positively associated with HbA_1c_ in those younger than age 40 years (0.04 percentage points higher HbA_1c_ per 5% increased increment in percent fat; 95% CI: 0.01 to 0.07; *P* < 0.01) but not associated in those age ≥40 years (0.03 percentage points higher HbA_1c_ per 5% increased increment in percent fat; 95% CI: −0.09 to 0.16; *P* = 0.6). Trunk percent was not significantly associated with HbA_1c_ in males with diabetes, although point estimates were consistent with the pattern of increased HbA_1c_ tracking with higher percent body fat (Table 2). Total lean mass was inversely associated with HbA_1c_ in males <40 years (0.04 percentage points lower HbA_1c_ per 5% increased increment in percent lean mass; 95% CI: −0.07 to −0.01; *P* = 0.01). No substantial associations were observed for total lean mass in males age 40 years and older nor between trunk lean mass and HbA_1c_ across age categories.

In females with diagnosed diabetes, only trunk but not total percent fat was significantly associated with glycemic control status. In this group, each 5% increase in trunk percent body fat was associated with a 0.05 percentage point higher HbA_1c_ (95% CI: 0.01 to 0.08; *P* = 0.006) for those <40 years and 0.11 percentage points higher HbA_1c_ for those age 40 years and older (95% CI: 0.06 to 0.17; *P* < 0.001). For lean mass, only trunk lean mass among females age 40 years and older was significantly associated with HbA_1c_ (0.05 percentage points lower HbA_1c_ per % increased increment of trunk lean mass; 95% CI: −0.08 to −0.01; *P* = 0.008); there was no association observed for total lean mass or trunk lean mass in females <40 years. The association of total trunk fat with HbA1c differed significantly by sex (*P* interaction < 0.001). The association of total and trunk percent fat as well as total lean mass with HbA1c differed significantly by age group (*P* interaction < 0.001).

We further examined the population without diagnosed diabetes to characterize the association of percent body fat and lean mass with likelihood (odds) of prevalent HbA_1c_ above the prediabetes lower cut point (≥5.7% or 39 mmol/mol, including those meeting criteria for undiagnosed diabetes; Table 3). Males younger than 40 years had 1.57 times the odds of HbA_1c_ ≥5.7% (39 mmol/mol) per 5% increase in total percent fat (95% CI: 1.28 to 1.93) and 1.50 times the odds of HbA_1c_ ≥ 5.7% (39 mmol/mol) per 5% increase in trunk percent fat (95% CI: 1.25 to 1.79). For males age 40 years and older, each 5% increase in total percent body fat was associated with 1.75 times the odds of HbA_1c_ ≥ 5.7% (39 mmol/mol, 95% CI: 1.53 to 2.00) and each 5% increase in trunk percent body fat was associated with 1.69 times the odds of HbA_1c_ ≥ 5.7% (39 mmol/mol, 95% CI: 1.50 to 1.91). Females younger than age 40 years had 2.01 times the odds of HbA_1c_ ≥ 5.7% (39 mmol/mol) per 5% increase in total percent fat (95% CI: 1.60 to 2.54) and 2.00 times higher odds of HbA_1c_ ≥ 5.7% (39 mmol/mol) per 5% increase in trunk percent fat (95% CI: 1.62 to 2.47). For females age 40 years and older, each 5% increase in total percent body fat was associated with 1.51 times the odds of HbA_1c_ ≥ 5.7% (39 mmol/mol, 95% CI: 1.37 to 1.65) and each 5% increase in trunk percent body fat was associated with 1.60 times the odds of HbA_1c_ ≥ 5.7% (39 mmol/mol, 95% CI: 1.47 to 1.74).

**Table 3.** **Adjusted Odds Ratios (95% CI) for the Association of Percent Fat and Lean Mass (Total Body and Trunk Per 5%) With the Presence of Prediabetes or Undiagnosed Diabetes (Hba_1c_ ≥ 5.7) Among Individuals Without Diagnosed Diabetes, NHANES 1999-2006**

	**Percent Total Body Fat**	***P* value**	**Percent Trunk Fat**	***P* value**	**Percent Total Lean Mass**	***P* value**	**Percent Trunk Lean Mass**	***P* value**
Male, y								
<40	1.57 (1.28–1.93)	<0.001	1.50 (1.25–1.79)	<0.001	0.63 (0.51–0.78)	<0.001	0.66 (0.55–0.80)	<0.001
≥40	1.75 (1.53–2.00)	<0.001	1.69 (1.50–1.91)	<0.001	0.56 (0.49–0.65)	<0.001	0.58 (0.52–0.66)	<0.001
Female, y								
<40	2.01 (1.60–2.54)	<0.001	2.00 (1.62–2.47)	<0.001	0.49 (0.38–0.62)	<0.001	0.49 (0.40–0.61)	<0.001
≥40	1.51 (1.37–1.65)	<0.001	1.60 (1.47–1.74)	<0.001	0.66 (0.60–0.73)	<0.001	0.62 (0.57–0.67)	<0.001

Adjusted for age, race/ethnicity, and height.

HbA_1c_ values in the prediabetes range (5.7% to 6.4%) or diabetes range (≥6.5%) combined into one group because of small sample size for undiagnosed diabetes.

Males younger than age 40 years had 0.63 times the odds of HbA_1c_ ≥ 5.7% (39 mmol/mol) per 5% increase in total percent lean mass (95% CI: 0.51 to 0.78) and 0.66 times the odds of HbA_1c_ ≥ 5.7% (39 mmol/mol) per 5% increase in trunk lean mass (95% CI: 0.55 to 0.88). For males age 40 years and older, each 5% increase in total percent lean mass was associated with 0.56 times the odds of HbA_1c_ ≥ 5.7% (39 mmol/mol, 95% CI: 0.49 to 0.65) and each 5% increase in trunk percent lean mass was associated with 1.69 times the odds of HbA_1c_ ≥ 5.7% (39 mmol/mol, 95% CI: 1.50 to 1.91). Females younger than age 40 years had 0.49 times the odds of HbA_1c_ ≥ 5.7% (39 mmol/mol) per 5% increase in total percent lean mass (95% CI: 0.38 to 0.62) and 0.49 times the odds of HbA_1c_ ≥ 5.7% (39 mmol/mol) per 5% increase in trunk percent lean mass (95% CI: 10.40 to 0.61). For females age 40 years and older, each 5% increase in total percent body fat was associated with 0.66 times the odds of HbA_1c_ ≥ 5.7% (39 mmol/mol, 95% CI: 0.60 to 0.73) and each 5% increase in trunk percent lean mass was associated with 0.62 times the odds of HbA_1c_ ≥ 5.7% (39 mmol/mol, 95% CI: 0.57 to 0.67).

## 3. Discussion

In this nationally representative sample of US adults age 18 to 69 years, total and trunk percent body fat were strongly associated with HbA_1c_ in those without diabetes. Among adults with diabetes, higher total percent fat was associated with higher HbA_1c_ in younger men age 18 to <40 years and higher trunk fat was associated with higher HbA_1c_ in females regardless of age. In the subgroup of adults with no history of diabetes, males were more likely to have elevated HbA_1c_ in the prediabetes or diabetes range (indicating undiagnosed diabetes) with both higher total and trunk percent fat across age ranges. Females also had higher odds of elevated HbA_1c_ with higher total and trunk percent fat, with the association strongest among women younger than 40 years of age. Furthermore, younger males with higher percent total lean mass and older females with higher percent trunk lean mass were more likely to have lower HbA_1c_ values. These results support prior evidence that demonstrates the importance of reduced fat mass in preventing and managing hyperglycemia [12, 14, 18, 27, 28].

Exercise intervention studies report that body composition, independent of weight loss, has a favorable impact on HbA_1c_. For example, the Health Benefits of Aerobic and Resistance Training in Individuals with Type 2 Diabetes (HART-D) trial—which randomized 201 adults with type 2 diabetes randomized to aerobic, resistance, or combined training for 9 months—found that changes in trunk fat mass and central adiposity were strongly associated with substantial reductions in HbA_1c_ over the 9-month intervention period [14]. A study by Sigal *et al.* randomized 251 adults with type 2 diabetes to aerobic training alone, resistance training alone, or a combined exercise training program [12]. The 22-week intervention yielded a similar improvement in glycemic control for participants receiving either aerobic or resistance training alone, with the greatest improvement among those receiving both aerobic and resistance training [12].

Several mechanisms provide insight into the role of body composition, a marker of “fitness,” as an independent contributor to glucose metabolism and insulin resistance compared with overall obesity, or “fatness.” Adipose tissue itself releases endocrine and bioactive mediators, in excess among persons with high percent body fat, which directly influences insulin resistance and hyperglycemia [29]. In particular, adipose tissue located in the trunk or central part of the body is more strongly associated with increased circulating glucose and insulin concentrations compared with peripheral parts of the body [7, 30]. For example, Bonora *et al.* compared fat topography in a population of obese and nonobese women and found that insulin resistance was more strongly correlated with central (abdominal) fat than with overall/nonabdominal fat [31].

Conversely, muscle mass plays a critical but often underappreciated role in glucose regulation and insulin action. Muscle works to clear glucose from plasma and plays a key role in glucose synthesis; disruption of glucose uptake by muscle directly contributes to the development of diabetes [32]. Although our measure of lean mass did not isolate muscle mass only, we were able to exclude bone mineral content and DXA provides a closer approximation of muscle mass than other more widely used approximations of body composition. Resistance training improves glucose control in patients with type 2 diabetes and increases disposal of excess circulating glucose [33]. Thus, although weight loss is often considered the first-line lifestyle target for those with type 2 diabetes and those at increased risk for diabetes, more active integration of resistance exercise would have direct impacts on both weight loss (and abdominal obesity) and glucose regulation. In the debate over the roles of “fitness vs fatness,” more attention should be paid to leveraging the known independent role of each component.

The strengths of this study include the availability of a large, nationally representative population of US adults both with and without diabetes. Additionally, the availability of body composition measures offers additional information above traditional measures of adiposity. A standardized protocol was also used to multiply imputed data sets for participants with missing body composition data, resulting in more complete analytic dataset.

Limitations of note include a lean mass measure in which muscle could not be specifically isolated. The measure of lean mass used includes all nonbone and nonfat components, both muscle as well as soft organ tissue, and therefore the proportion of lean mass serves as a proxy for muscle mass. However, this measure is more specific than BMI, for example, which assumes a constant proportion of muscle to overall mass. Additionally, the study population of younger adults with diabetes was small. Nonsignificant associations in this group may at least partially be driven by the limited sample size in these groups but also potential competing factors in this patient population. Finally, the cross-sectional nature of the study precludes the study of the temporal relationship between body composition and hyperglycemia.

Taken together, prior evidence along with the current study emphasizes the importance of body composition in additional to overall adiposity as targets for prevention and management of hyperglycemia. In this population-based study, the association was strongest among individuals without established diagnosed diabetes, though substantial associations were observed for trunk (central) percent fat among women with diagnosed diabetes. Additionally, among adults with no history of diabetes diagnosis or treatment and both total and trunk percent fat were significantly associated with increased likelihood of elevated HbA_1c_ values in the prediabetes range or above the clinical cut points for diabetes. Interventions that target both weight loss where warranted and decreasing the proportion of fat in relation to lean mass via resistance training may have the most beneficial impact particularly for diabetes prevention.
